# Kinetics, energy efficiency and mathematical modeling of thin layer solar drying of figs (*Ficus carica* L.)

**DOI:** 10.1038/s41598-021-00690-z

**Published:** 2021-10-28

**Authors:** Lahcen Hssaini, Rachida Ouaabou, Hafida Hanine, Rachid Razouk, Ali Idlimam

**Affiliations:** 1 National Institute of Agricultural Research (INRA), Rabat, Morocco; 2 Department of Chemistry, Faculty of Science Semlalia, Cadi Ayyad University, B.O. 2390, 40000 Marrakesh, Morocco; 3 Laboratory of Bioprocess and Bio-Interfaces, Faculty of Science and Technics, BO 523, Beni-Mellal, Morocco; 4 Laboratory of Solar Energy and Medicinal Plants, Teacher’s Training College, Cadi Ayyad University, BP 2400, Marrakesh, Morocco

**Keywords:** Biophysics, Plant sciences

## Abstract

First convectional thin layer drying of two fig (*Ficus carica* L.) varieties growing in Morocco, using partially indirect convective dryer, was performed. The experimental design combined three air temperature levels (60, 70 and 80 °C) and two air-flow rates (150 and 300 m^3^/h). Fig drying curve was defined as a third-order polynomial equation linking the sample moisture content to the effective moisture diffusivity. The average activation energy ranged between 4699.41 and 7502.37 kJ/kg. It raised proportionally with the air flow velocity, and the same patterns were observed for effective moisture diffusivity regarding drying time and velocity. High levels of temperature (80 °C) and velocity (300 m^3^/h) lead to shorten drying time (200 min) and improve the slices physical quality. Among the nine tested models, Modified Handerson and Pabis exhibited the highest correlation coefficient value with the lowest chi-square for both varieties, and then give the best prediction performance. Energetic investigation of the dryer prototype showed that the total use of energy alongside with the specific energy utilization (13.12 and 44.55 MWh/kg) were inversely proportional to the velocity and drying temperature. Likewise, the energy efficiency was greater (3.98%) in drying conditions.

## Introduction

Figs (*Ficus carica* L.) are important source of essential dietary nutrients such as fibers, carbohydrates, minerals and vitamins^1^. Since the moisture content of fresh figs is, generally, more than 80%, they are classified as highly perishable. Keeping the fruits fresh, has always been the best way to maintain their health promoting compounds, but most storage techniques require low temperatures, which are difficult to maintain throughout the distribution chain^2^. However, even in low temperature or refrigerated conditions, figs may not be stored for a long period, and thus maintain their nutritional value. Traditionally, many preservation technics of fruits have been applied since generations ago like salting, dehydration and fermentation^3^. Drying is among the most appropriate food preservations means that is still being widely practiced until today. Drying has been recognized as a promising alternative to extend figs shelf-life and for postharvest management, as figs are among the world's largest dried crops^4,5^. It is also one of the apparent technics to increase its production to meet the market demand by reducing wastage, especially in undeveloped countries where exist poorly established logistics for low temperature distribution and handling facilities^6^. Drying can be processed using several solar drying technics.

Numerous studies dealt with innovative indirect solar dryer, such as the photovoltaic-thermal hybrid solar reported by Slimani et al.^7^, where the results showed the effectiveness of the used configuration, with low electrical power requirements. In the study of Vijayan et al.^8^, a dryer prototype combined with porous sensible heat storage medium, was investigated for drying bitter gourd, which was more consistent and produced better quality product as compared to open sun drying. Likewise, Chaouch et al.^9^, used solar dryers with different principles to study camel meat drying with very satisfactory final quality of dried camel meat with better preservation of protein rate of 83.5%. The indirect solar convective drying may become a more convenient alternative for small exploitations in rural sector and other areas where electricity is scarce and in regular supply^3^. Likewise, it can reduce wastages, improve dried products quality and is economically advantageous compared to traditional drying methods^6^. Furthermore, several problems which are associated with open-air drying can be solved by using a solar dryer^10^.

Drying depicts a complex process of heat and mass transfer of removing moisture from biological products. In food processing, the main role of drying technologies is to preserve agricultural commodities quality, extend their shelf life and produce new products, that would not otherwise be feasible^3^. Furthermore, drying allows to obtain a desired moisture content, physical form, flavor, color, or texture, and to reduce the volume or the weight for low cost transportation^11^. Using drying is technically convenient and the cost associated for processing, packaging, transportation, and storage is sensitively less for dried fruits than canned and frozen products^12^. The simulation of drying experimental results with help of empirical and semi-empirical models is valuable for designing new or in improving existing drying systems. They are also helpful in drying process configuration and control^13^. In food drying kinetics, the constant K is used instead of transport proprieties (thermal diffusivity, thermal conductivity and moisture diffusivity), that describes the drying process. Simply put, the constant K combines all the transport properties and defined the drying process. Numerous thin layer equations exist in the literature and are useful for investigating drying of several agricultural products, such as, apricot^14^, mullbery^15^, Strawberry^13^, eggplant^16^, citrus^17^, banana^18^ and cherry^19^. These models were useful in describing the drying kinetics of numerous products under different aerothermal conditions and to estimate their drying time. They also serve to upsurge the drying efficiency and to generalize drying curves for the design and operation of dryers.

Nowadays, figs are an important crop worldwide with is an increasing interest in their consumption either fresh or dried as they are an important component for highly nutritive diet^20^. Morocco ranks 3rd in the world’s fig production with about 153,472 tons, representing about 8% of the worldwide fig production, after Turkey (310,000 tons) and Egypt (225,295 tons)^21,22^. The total production of these top three fig producers accounts for more than 52% of the world’s total^23^. Turkey is the first fig exporter worldwide with about 41.6% of the world’s total. Most of these exportations concerns dried figs with an export value of 29,291,000 $, representing about 31.6% worldwide^23,24^.

Most of the Moroccan fig production is concentrated in the northern mountainous regions, where its cultivation is the main agricultural activity after olive (*Olea europaea*). However, because of the poor postharvest infrastructure and handling facilities needed to maintain product freshness and nutritional value, figs were, until recently, considered as a secondary crop and have been underexploited. Nowadays, an important interest was given to solar drying technics, since Morocco receives an intensive daily average solar radiation intensity of 5.3 kW h/m^2^ and 5 to 6 h of sunshine duration in the winter and 9 to 10 h during the summer^25^. The exploitation of this abundantly available source of energy could be very useful in most of Moroccan regions, particularly during summer and can be used for many applications such as drying, heating, distilling water, etc.^26^. This makes solar thermal application for agricultural products drying, particularly figs to be integrated. Furthermore, while a few numbers of studies have been published over the last decade on drying of some Moroccan crops like prickly pear^27^, sweet cherry^19^, the conducted literature review, revealed that no similar studies have been carried out on figs. In this context, two fig varieties growing in Morocco, namely, ‘Rey Blanche’ and ‘Conidria’ were studied for their drying behavior using partially indirect convective dryer. This study aims to : (1) study the fig drying kinetics using two of the most cultivated varieties in Morocco, (2) investigate the effect of drying temperature and velocity on the fig drying kinetics, (3) determine the characteristic drying curve (CDC) of each fig variety, (4) calculate the effective diffusivity and the average activation energy, (5) fit the fig drying curves using several semi-theoretical and theoretical models in order to select the one providing the highest prediction throughput resolution, and to (6) determine the specific thermal consumption and overall thermal efficiency of the dried used.

This work contributes to a detailed research and information about figs drying proprieties and modeling growing in Morocco. In the present study is the first systematic investigation of thin layer behavior in figs during hot air forced convection, alongside with the energy consumption and efficiency in a solar dryer under the Moroccan conditions. It, therefore, investigates, the possibility of adopting forced convection solar drying to reduce post-harvest losses of figs, harvesting and optimizing free solar energy, and helping local farmers economically. The herein proposed solar dryer prototype can be manufactured locally; cheaply, thus it will be totally affordable by smallholder farmers along with traditional cooperatives clusters. This prototype can have wide application especially in remote mountainous areas as well as in the south area where sunshine is abundant during the whole year.

## Materials and methods

### Preparation

Fresh figs belonging to ‘Rey Blanche’ and ‘Conidria’ varieties planted in the ex-situ collection the National Institute for Agricultural Research (INRA) in Meknes, Morocco. They were harvested at their full repining during August of 2019. The experimental research complies with IUCN Policy Statement on Research Involving Species at Risk of Extinction and the Convention on the Trade in Endangered Species of Wild Fauna and Flora. The fruits were kept in polyethylene bags and transported in portable cooler. The figs initial moisture was determined by the standard gravimetric method at 105 °C for 24 h according to AFNOR NF V03-40. Each measurement was performed in triplicate.

### Drying experiments

The samples were cut in thin slices of 70 g following a uniform thickness of ~ 6 mm using ceramic knife. The experimental design combined three air temperatures levels (60, 70 and 80 °C) and two drying velocity levels 150 and 300 m^3^/h. The dryer was started about 40 min before drying experiments to achieve steady-state conditions. The fig slices were spared uniformly on the first rack of stainless-steel mesh (mesh size 10 × 10 mm) to ensure homogeneity of diffusion during the process. The heated air goes through the drying chamber trays, from the bottom and flowed upwards to carry out the samples’ moisture. The auxiliary heater served for controlling and keeping the air temperature constant.

The fig slices wet Mh(t) was monitored through time using a precision balance (± 0.01 g). Initially, the samples weighing *M*_*h*_*(t)* was performed every 5 min for the first hour, then increased to 10 min for the second hour and 20 min until constant weight. Dry weight *M*_*d*_ was measured after each experiment by keeping samples at 105 °C for 24 h. The moisture content was determined using Eq. (1). The temperature and air humidity at drying entrance unit were measured using a HI 9564 Thermo Hygrometer (Hanna Instruments Ltd, Bedfordshire, UK).

1 $$ M\left( t \right) = \frac{{M_{h} \left( t \right) - M_{d} }}{{M_{d} }} $$

### Experimental set up

**Figure 1:**
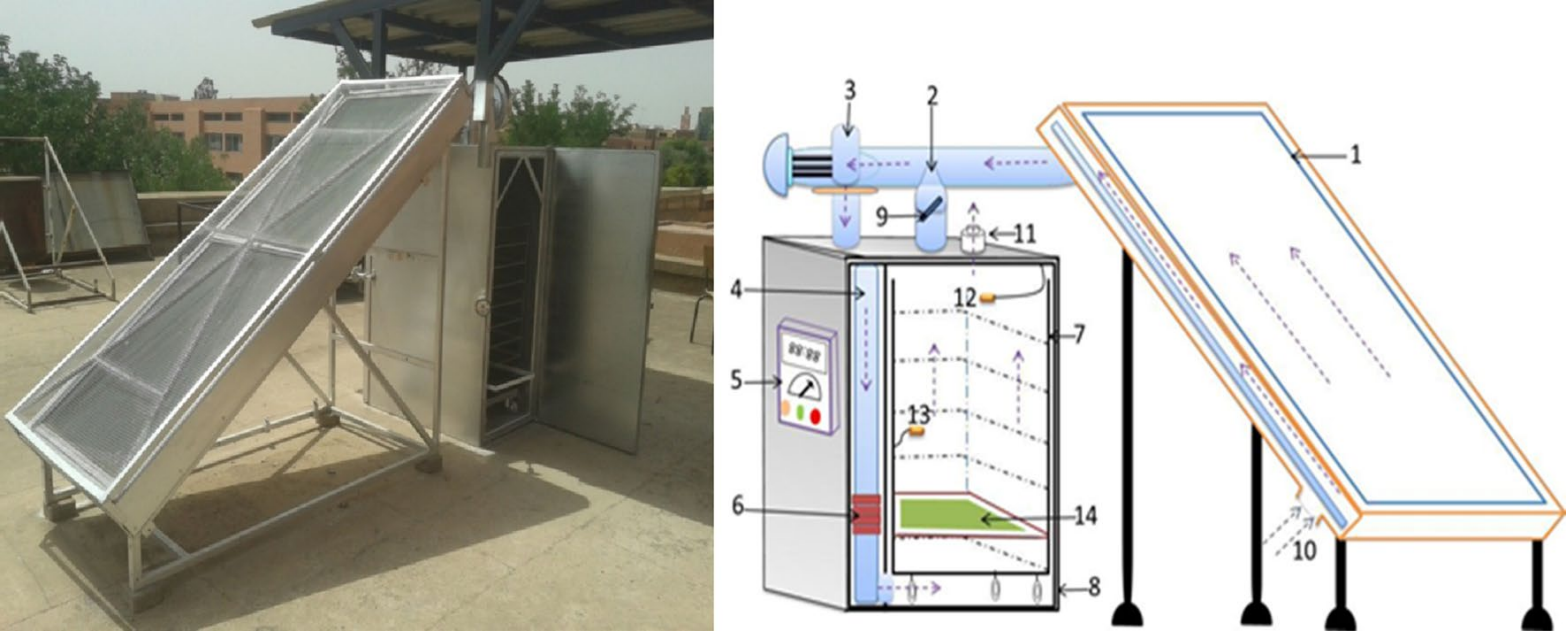
Schematic diagram of the solar dryer. (1) Solar collector, (2) circulation fan, (3) fan, (4) air flow direction, (5) control box, (6) auxiliary heating system, (7) shelves, (8) drying cabinet, (9) recycling air, (10) control foot, (11) exit of air, (12) humidity probes and (13) thermocouples (14) sample holder (Ouaabou et al.^19^).

Figs slices were dehydrated in hybrid convection solar dryer (Fig. 1). The prototype produces 80% of solar radiation, and is basically composed of four main parts that are: solar air collector, drying chamber, circulation fan and auxiliary heater. The solar collector was a 10° inclined black galvanized sheet iron with dimensions of 1 m by 2.5 m and a thickness of 0.5 mm. It was a non-selective surface-oriented southward with a single circulation and glazed. The drying chamber was 1.40 m of height, 0.5 m width, and 0.90 m depth and has 10 racks. A centrifugal fan (0.0833 m^3^/s; 80 mm CE, 220 V) provides a theoretical velocity of 1.7 m/s with a regulator which allows to fixed the air flow rate within a range of 150 to 300 m^3^/h. The circulation fan that supplies fresh air has a power of 0.1 kW. The auxiliary heater has a power of 4 kW. It was connected to a thermoregulator which allows to set the temperature (0 to 99 ± 0.1 °C) of drying chamber. Further details regarding accuracy of different drying compartments alongside that of other devices herein are summarized in the Table 1.

**Table 1:** Estimated uncertainties for different measurements.

Measured property	Uncertainty	Range
Inlet air temperatures by a thermo-regulator connected to a PT100 platinum probe acting on the electric auxiliary heater	± 0.1	0–100 °C
Inlet and outlet air humidity (relative humidity sensor) by ABS body RH probe with built-in microchip, °C/°F temperature readout 250-h battery life with a battery level indicator	± 3	0 to 100%
Flow rate (A centrifuge fan)	± 1.0	0.028 to 0.083 m^3^ s^−1^
Ambient air temperature measurement by HI 935,005 K-Thermocouple probe °C/°F temperature readout 200-h battery life with a battery level indicator	± 0.2	0–100 °C
Sunlight measurement by Solar Power meter; Data hold function to hold measurement values High accuracy and rapid response	± 10	up to 1999 W/m^2^ or 634 BTU/(ft^2^*h)
Weigh sample by Digital weighing device, RS232 Interface Glass wind-shield	± 0.1	0–5 kg

*BTU* British thermal unit; *ft* foot; *W* Watt; *ABS* absolute humidity; *HR* relative humidity.

### Determination of moisture ratio and dimensionless drying rate

The final moisture content measurement is critical. It helps to investigate the drying progression and determines whether the process was accomplished or not. This variable is a great indicator on the sample physicochemical quality. The moisture ratio (MR) and the dimensionless drying (f: ratio of sample initial drying rate and drying rate at a specific time of the experiment) of samples were determined as follow:

2 $$ {\text{MR}}\left( t \right) = \frac{{M\left( t \right) - M_{e} }}{{M_{0} - {\text{M}}_{e} }} $$

3 $$ {\text{f = }}\frac{{\left( { - \frac{{{\text{dM}}}}{{{\text{dt}}}}} \right)_{t} }}{{\left( { - \frac{{{\text{dM}}}}{{{\text{dt}}}}} \right)_{0} }} $$

where *M(t)* is the sample moisture content versus time (kg water/kg dry weight), *M*_*0*_ is the initial water content (kg water/kg dry weight), *M*_*e*_ is equilibrium moisture content of fig samples (kg water/kg dry solid), $\left( { - \frac{dM}{{dt}}} \right)_{0}$: initial drying rate, (kg water/(kg dry matter.min)) and $\left( { - \frac{dM}{{dt}}} \right)_{t}$: drying rate at a specific drying time (kg water/(kg dry matter.min)).

The determination of the drying kinetics is achieved using appropriate software calculation (DOS Smoothing, Curve Expert 3.1 and Originpro 8.1). The drying rate corresponding to each experiment was calculated using Lissage software under MS-DOS.

### The effective moisture diffusivity

The effective moisture diffusivity D_eff_ is an important transport property in food drying processes modeling. It indicates the flow of moisture within the drying product. Moisture migrates from the inside of the product and reaches the outer surfaces under the action of various moisture transport mechanisms that can be combined (i.e. capillary flow, Knudsen diffusion, surface diffusion, evaporation and condensation, pure diffusion, ect.). This moisture thereafter, is evaporated through the air due to convection^28^. Fick’s equation might be performed to depict the drying behavior through falling rate period. It is presented in Eq. (4):

4 $$ \frac{\partial MR}{{\partial t}} = Deff \nabla^{2} \;\;MR $$

Assuming that the transport of moisture carries out by diffusion, the shrinkage is neglected, the particle is homogenous and isotropic, initial moisture and temperature distribution are uniform, the analytical solution of Fick's can be developed as shown in Eq. (5) ^29^.

5 $$ MR = \frac{8}{{\pi^{2} }}\mathop \sum \limits_{n = 1}^{\infty } \frac{1}{{n^{2} }} \exp \left( { - \left( {2n + 1} \right)^{2} \pi^{2} \frac{{D_{eff} t}}{{R^{2} }}} \right) $$

For a sufficiently long drying period, the above equation becomes^30^:

6 $$ \ln \left( {MR} \right) = \ln \left( {\frac{8}{{\pi^{2} }}} \right) - \left( {\frac{{\pi^{2} {\text{D}}_{{{\text{eff}}}} {\text{t}}}}{{4{\text{L}}^{2} }}} \right) $$

where L (in m) is the half-thickness of the used samples.

D_eff_ could be determined through the slope method. Indeed, Eq. (6) is transformed into Eq. (7):

7 $$ D_{eff} = - \frac{{B4L^{2} }}{{\pi^{2} }} $$

The *D*_*eff*_ may be deduced from the slope of Eq. (8) by fitting the drying experimental data. The activation energy *E*_*a*_, is the lowest energy level (minimum), that must be exceeded for this process to occur. The E_a_ value is related to *D*_*eff*_ and its dependence on temperature is expressed by Arrhenius model^11^. The self-diffusion origin is closely related to the thermal agitation. Afterword, the diffusion is activated, and the *D*_*eff*_ is calculated following the Arrhenius law as shown in the Eq. (8):

8 $$ D_{eff} = D_{0} \exp \left( { - \frac{{E_{a} }}{RT}} \right) $$

where *D*_*0*_ is the Arrhenius law pre-exponential factor (m^2^ s^−1^), *E*_*a*_ is the activation energy (kJ mol^−1^), *R* is the perfect or ideal gas constant (8.314 kJ mol^−1^), and *T* (in K) is the air temperature^31^.

The activation energy is calculated by plotting the ln(D_eff_) as a function of the reciprocal of the temperature $\left( {\frac{1}{{\text{T}}}} \right)$.

### Drying curves modeling

To describe and predict the fig thin-layer drying kinetics and model its moisture content ratio, nine mathematical models were used (Table 2). The experimental data were fitted using nine empirical and semi-empirical mathematical models (Table 2). Fitting robustness was evaluated based on to the following criteria:

- highest coefficient of correlation (r);
- the lowest reduced chi-square (*χ*^2^).

**Table 2:** Mathematical models applied to fig drying curves.

Model	Equation	References
Lewis	MR = exp(-kt)	Robert et al.^32^
Page	MR = exp(-kt^n^)	Singh et al.^33^ Aghbashlo et al.^34^
Logarithmic	MR = aexp(-kt) + c	Kingsly et al.^35^
Wang and Singh	MR = 1 + at + bt^2^	Babalis et al.^36^
Diffusion approach	MR = aexp(-kt) + (1-a)exp(-kbt)	Wang and Singh^37^
Midilli-Kucuk	MR = aexp(-kt^n^) + bt	Akpinar et al.^38^
Handerson and Pabis	MR = aexp(-kt)	Akpinar et al.^39^
Modified Handerson and Pabis	MR = aexp(-kt) + bexp(-gt) + cexp(-ht)	Goula et al.^40^
Two term	MR = aexp(-k_1_t) + bexp(-k_2_t)	Henderson and Pabis^41^

These statistical parameters are defined by:

$$ r = \sqrt {\frac{{\mathop \sum \nolimits_{i = 1}^{N} \left( {MR_{{eq_{exp,i} }} - \overline{MR}_{{eq_{exp,i} }} } \right)^{2} }}{{\mathop \sum \nolimits_{i = 1}^{N} \left( {MR*_{{eq_{exp,i} }} - \overline{MR} *_{{eq_{exp,i} }} } \right)^{2} }}} $$

$$ \chi^{2} = \frac{{\mathop \sum \nolimits_{i = 1}^{N} \left( {MR_{pre,i} - MR_{\exp ,i} } \right)^{2} }}{N - n} $$

where MR*_pre,i_: i^eme^ Moisture ratio predicted by model; MR*_exp,i_: i^eme^ Experimental moisture ratio; N: Number of experimental data; n: Number of constants.

### Energy consumption of the solar dryer

The total energy consumed (kWh) of convective solar dryer was obtained using Eq. 6^42^.

9 $$ E_{t} = \left( {A.v.\rho_{a} .C_{a} .\Delta T.D_{t} } \right) + E_{mec} $$

where E_t_ is the total energy consumption (kWh) of drying system, *A* is the tray area (m^2^), ν is the air velocity (m/s), *ρ*_*a*_ is the air density (kg/m^2^), *C*_*a*_ is the air specific heat (kJ/kg °C), ΔT is the temperature difference (°C), *D*_*t*_ is the time of the entire drying process (h) and ρ_a_ is the microwave power (kW)^42^.

The inlet heat capacity^43^ and microwave power^44^ were calculated using Eqs. (7) and (8):

10 $$ \rho_{a} = \frac{101.325}{{0.287 \times T}} $$

11 $$ C_{a} = 1.04841 - \frac{3.83719 \times T}{{10^{4} }} + \frac{{9.45378 \times T^{2} }}{{10^{7} }} + \frac{{5.49031 \times T^{3} }}{{10^{10} }} + \frac{{7.92981 \times T^{4} }}{{10^{14} }} $$

E_mec_ is the mechanical energy (consumed by the fan and the auxiliary heater) used during each drying experiment and measured by the mean of an electric energy meter (accuracy of 0.01 kWh).

The specific thermal energy (SEC (kWh/kg)) needed to remove 1 kg of moisture from the fig sample was calculated using Eq. 12^45^

12 $$ SEC = \frac{{E_{t} }}{{m_{w} }} $$

where m_w_ is the mass of removed water (kg) and was calculated using Eq. 13^46^:

13 $$ m_{w} = \frac{{W_{0} \left( {Y_{0} - Y_{f} } \right)}}{{100 - Y_{f} }} $$

where W_0_ is the initial weight of sample (kg), Y_0_ refers to the initial moisture content (% d.b) at time (t = 0) and Y_f_, the sample final moisture content (% d.b).

### Color and water activity determination

The L*, a* and b* color coordinates were determined before and after each drying experiment using a NH310 colorimeter (Shenzhen 3NH Technology, China)^47^. The colorimeter was calibrated to a white calibration plate. The peels color measurements were obtained from two spots located on opposite sides of the slice diameter, while the pulp color was determined from two arbitrary spots on the both sides of the slice. The mean of the two measurements was considered as one replicate. Color differences of fig peels and pulp between the dry and fresh samples were used to describe the color change during drying, defined using Eq. (14), as follows:

14 $$ \Delta E = \sqrt {\left( {L_{0}^{*} - L^{*} } \right)^{2} + \left( {a_{0}^{*} - a^{*} } \right)^{2} + \left( {b_{0}^{*} - b^{*} } \right)^{2} } $$

where L*represents on the scale CIELAB the lightness of the sample ranging from 0 (black) to 100 (white), coordinate a* represents red color ( +) or green (−), and coordinate b* represents yellow color ( +) or blue (−). Subscript 0 refers to the color of the fresh fig slices. High DE values indicate large color changes compared to the reference. Water activity of fresh and dried slices was assessed by a calibrated electric hygrometer (HygroLab, Rotronic, Bassersdorf, Switzerland). All measurements were carried out in triplicates.

### Statistical analysis

The results herein reported are expressed as means ± SE (standard error). Analysis of variance was performed by GLM procedures (SPSS 22 for Windows). The *p* value < 0.05 was considered statistically significant.

## Results and discussions

**Figure 2:**
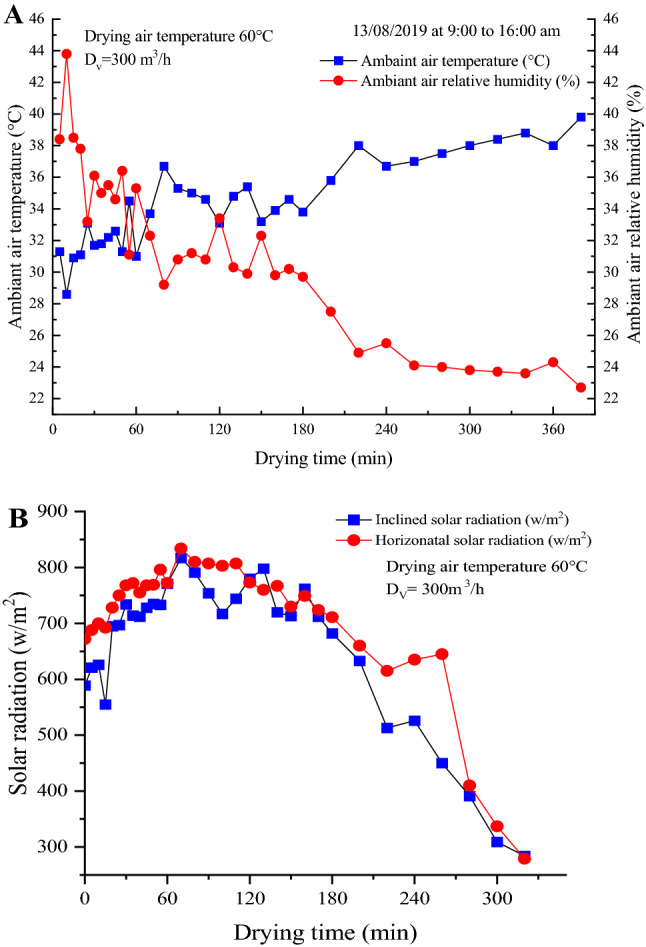
Air relative humidity, temperature (**A**) and solar radiation (**B**) on the dryer collector during the experiment of drying air temperature of 60 °C.

All experiments started at 9:00 a.m. and continued till 6:00 p.m., during which the city receives the maximum of solar radiation. Figure 2 shows data of the ambient air temperature, relative humidity and solar radiation for fig drying experiment at 60 °C and velocity of 300 m^3^/h. The temperature of the ambient air was found between 30 and 40 °C, while the ambient air humidity varied between 23 and 44%. Horizontal solar radiation was slightly higher than the inclined. Thus, the values ranged from 280 to 850 W/m^2^ and 280 to 830 W/m^2^, respectively. The initial moisture content of the studied samples (wet basis) was 78 ± 1% for ‘Rey Blanche’ and 75 ± 1.2% for ‘Conidria’. This initial moisture was reduced for both samples to a final moisture content of 23 ± 1%.

### Drying curves

**Figure 3:**
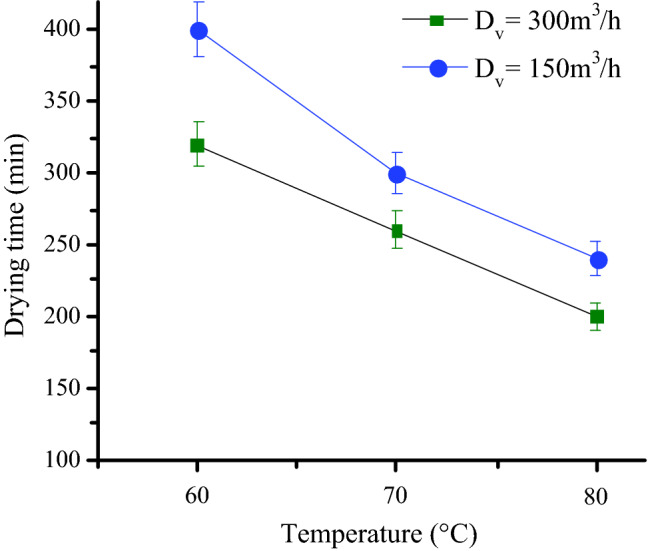
Drying time as a function of temperatures and drying volume flow rate.

Drying time was determined by jointly using drying temperature and velocity variables. It defines the time required to lower the moisture content of samples to the moisture content suitable for their conservation. The drying time of fig slices for different aero-thermal conditions is given in Fig. 3. According to the results, drying temperature and volume velocity displayed a significant effect on drying time, particularly due the osmotic pressure increase. Hence, at a constant air velocity, arising the air temperature from 60 to 80 °C decreased significantly the drying time. Also, at a constant air temperature, increasing air velocity from 150 to 300 m^3^/h decreased substantially the drying time, as a result of increasing convective heat and mass transfer coefficient between the drying air and the samples. For almost all agricultural products, the drying process took place in the falling rate period where the water molecule is strongly linked to the structure of the product; therefore, the effect of the drying temperature is more important comparatively to the air velocity. Indeed, drying temperature of 80 °C coupled to air flow rate of 300 m^3^/h, provide the optimum condition for fig slices drying in a shortened period of 200 min. These results are consistent with those reported by Garba et al.^48^, Ouaabou et al.^19^ and Bahammou et al.^49^, who attributed the great impact on drying kinetics, primary to the temperature and then velocity.

**Figure 4:**
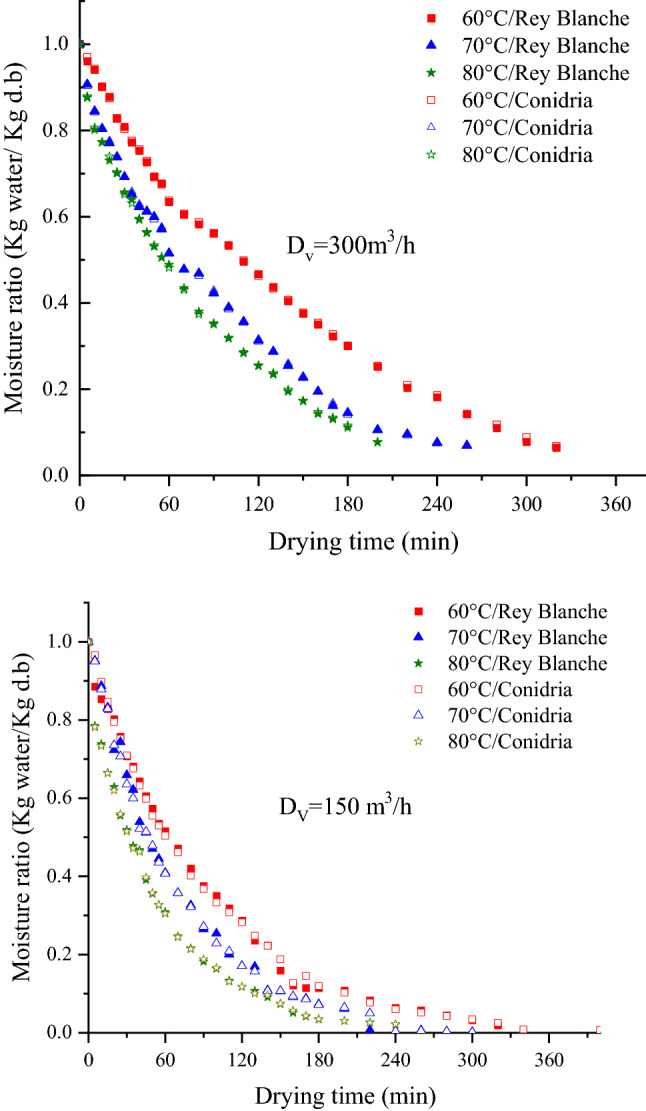
Experimental curves of fig moisture content as a function of time, at various air temperatures (60, 70 and 80 °C) and drying volume flow rate (150 and 300 m^3^/h).

As observed from Fig. 4, the drying curves showed a sharp decrease of moisture content as the drying time increases. The figure shows an absence of phase 0, period of the increasing drying-rate, where the sample temperature increased without any significant loss of moisture, and phase I, known as the period of constant drying-rate. Therefore, only the falling drying rate period (phase II) was observed. This may be due to the small difference between the wet air temperature and the sample initial temperature. These results are in agreement with previous reports^19,48–51^. Obviously, increasing the drying temperature implies an important rise of the drying rate, and thus a substantial decrease of the drying time. It is noteworthy that the drying flow rate of 300 m^3^/h leaded to sharp decrease of the moisture content and drying time shortening. Similar pattern was observed at a low flow rate (150 m^3^/h), but the drying time was lengthened. The varieties ‘Rey Blanche’ and ‘Conidria’ showed approximately the same behavior to different air-drying conditions on account of small difference in their initial moisture. In general, the time needed to obtain the targeted moisture content to any given level was dependent on the aerothermal factors, being the highest at the temperature of 60 °C and lowest at 80 °C. Thus, for both varieties, the drying time at Dv = 300 m^3^/h varied from 200, 260 to 360 min at 80, 70 and 60 °C, respectively, whereas, at 150 m^3^/h, the drying time was 240, 300 and 400 min at air temperature of 80, 70 and 60 °C, respectively. At a constant drying flow rate, the drying time was decreased for 70% and 60%, as the temperature increased from 60 to 80 °C, respectively for 300 and 150 m^3^/h. On the other hand, decreasing the air flow rate from 300 to 150 300 m^3^/h, the drying time was lengthened by 26 and 16% at 60 and 80 °C, respectively.

### Influence of temperature and air flow rate

**Figure 5:**
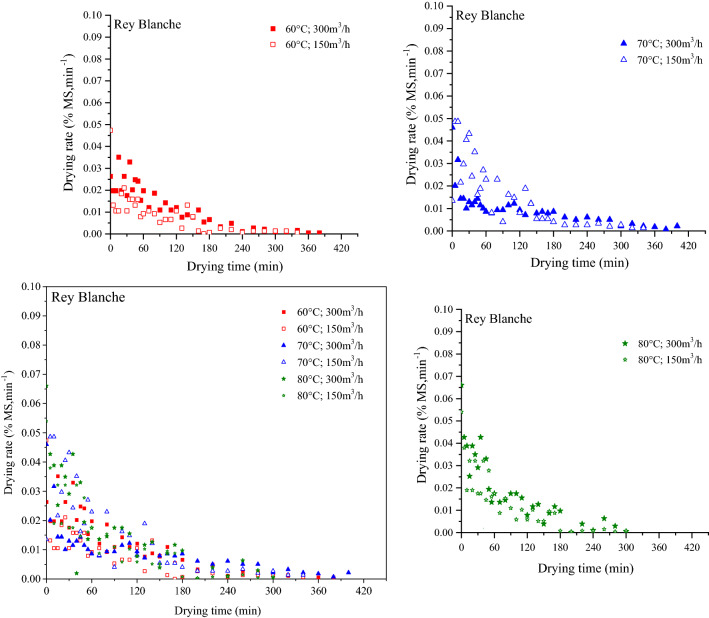

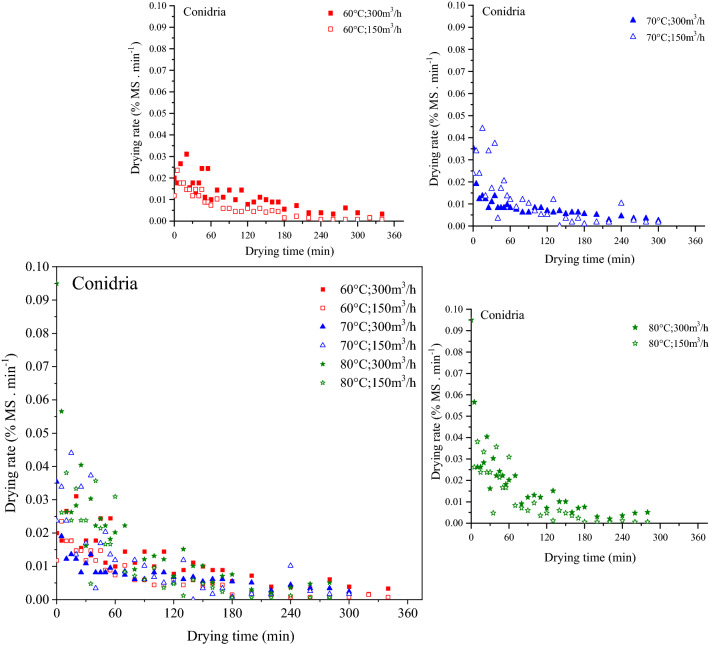
Drying rate of fig slices as a function of drying time. Curves for each temperature are embedded separately.

The influence of temperature and air flow rate on drying rate was shown in Fig. 5. In fact, the samples show that drying rate took place only in the falling rate period and no constant stage was noticed. It was observed that, for the same air flow rate, drying rate of fig slices increased as drying temperature increased. Consequently, the drying kinetics of fig is particularly linked to the variation in drying air temperature. Comparable findings were reported by Akanbi et al.^52^ for tomato slices, Krokida et al.^53^ for some different vegetables and Doymaz^15^ for thin carrot and mulberry and Doymaz^30^ for figs. This implies that no water film did exist at the exterior of the fig slices and that the moisture transfer from the interior of the samples to its surface occurred under the action of several complicated transport mechanisms (liquid diffusion or vapor diffusion or capillary forces). Those mechanisms change during the drying process^54^. So far there is no theory which explains the real mechanism behind moisture transfer in the falling drying rate. Nevertheless, diffusion remain the only probable mechanism which describes the mass transfer in agri-foods^31^. Indeed, there was no difference in drying rate between the two fig varieties. Similar result was reported in other studies^19,55,56^.

### Change in physical and color characteristics

**Figure 6:**
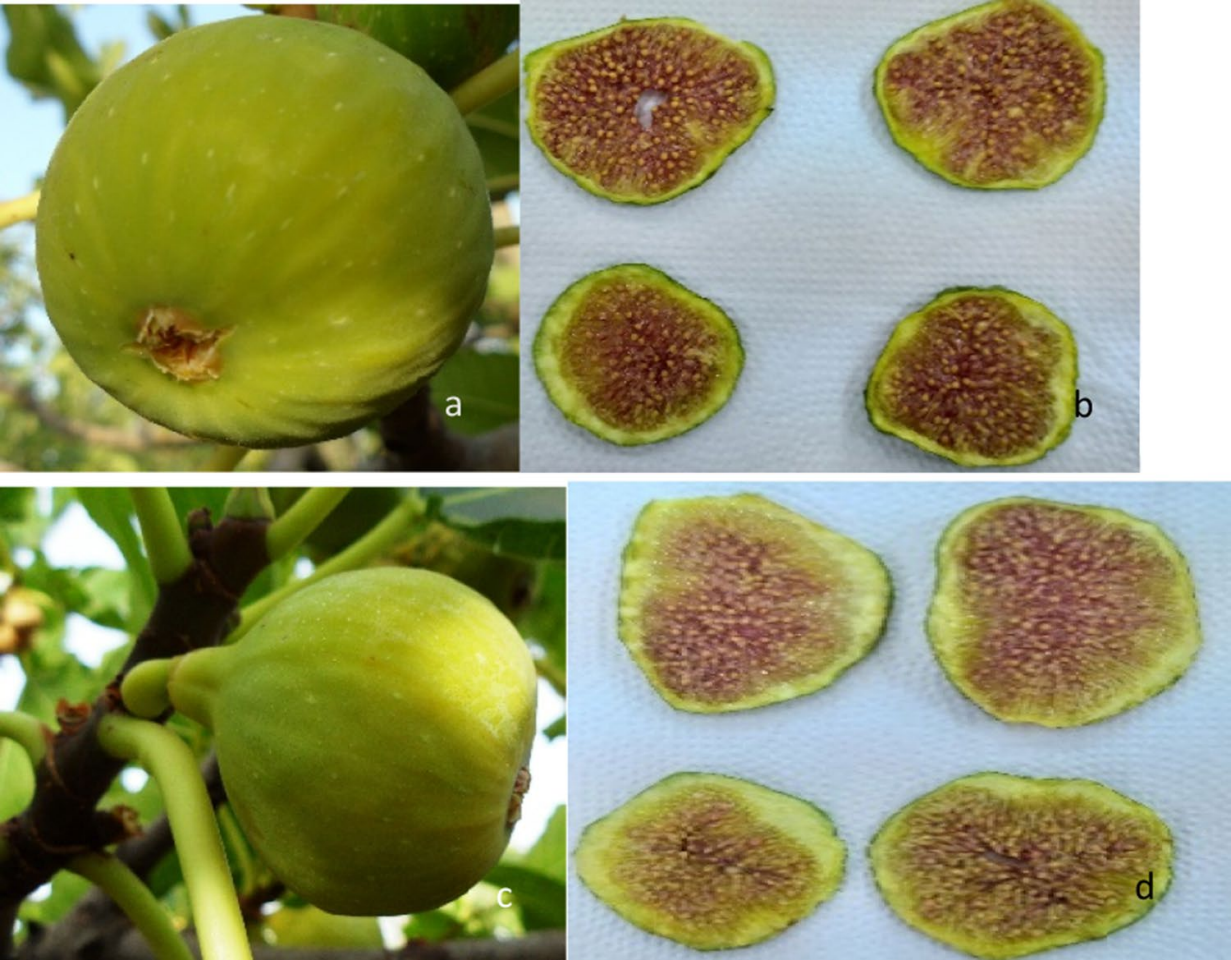
Pictures of the whole fruits and their dried slices at 80 °C and 300 m^3^/h. (**a**–**d**) refer to ‘Conidria’ and ‘Rey Blanche’, respectively.

Figure 6 shows the photos of fig samples in the fresh state and the aspect of their dried slices at 80 °C and 300 m^3^/h. Both varieties showed significant impact of temperature and drying air-drying flow rate (D_v_) on their physical traits. Slice thickness, water activity (a_w_) and total color difference (ΔE) was used as a parameters of dried fig quality (Table 3). The Aw of dried fig water activity, is important to the quality and stability of the product. It is a determinant for the growth of microorganisms and related as well to degradation reactions of a chemical, enzymatic, and physical nature. A_w_ of dried samples varied from 0.3 to 0.46 which fell within the standard a_w_ of dried food^57^. Fig slice thickness were significantly reduced by temperature. The values varied, generally, between 2.6 and 4.15 mm. The total color difference during thin layer drying is also an important indicator of the dried product quality. It indicates the browning reaction reactions linked to the color change that could occurred during drying period^58^. According to the Table 3, ΔE of both peels and pulp decrease significantly when temperature and D_v_ increase. It is noteworthy that all these parameters were significantly higher at low temperature and air flow rate (D_v_). Therefore, best physical conditions of figs’ slices conservation were obtained at high temperature and air-drying flow rate (80 °C and 300 m^3^/h, respectively). Table 4 shows a highly significant impact of temperature and D_v_ on the quality of samples. However, both varieties displayed similar behavior an then no statistically significant difference was spotted. Furthermore, the interaction between temperature, D_v_ and variety was statistically significant.

**Table 3:** Physical traits of dried fig slices as a function of temperature and drying air flow rate and temperature.

			Water activity (a_w_)	Thickness (mm)	ΔE (Peels)	ΔE (Pulp)
Dv = 150 m^3^/h	60 °C	Rey Blanche	0.44 ± 0	4.01 ± 0.46	44.26 ± 0.7	37.51 ± 4.2
	70 °C		0.44 ± 0	3.85 ± 0.52	33.54 ± 1.3	35.24 ± 3.6
	80 °C		0.34 ± 0.01	3.47 ± 0.39	30.1 ± 3	31.91 ± 3.6
	60 °C	Conidria	0.46 ± 0.02	4.1 ± 0.18	50.7 ± 2.6	44.13 ± 1.8
	70 °C		0.4 ± 0.01	3.93 ± 0.31	48.8 ± 3	35.67 ± 0.7
	80 °C		0.35 ± 0.1	4.13 ± 0.64	44.69 ± 0.8	32.06 ± 1.4
Dv = 300 m^3^/h	60 °C	Rey Blanche	0.41 ± 0.01	4.15 ± 0.47	46.76 ± 1.6	43.91 ± 3
	70 °C		0.36 ± 0.01	3.76 ± 0.72	43 ± 2.3	40.36 ± 1.9
	80 °C		0.3 ± 0	2.9 ± 0.7	22.09 ± 1.1	29.77 ± 2.9
	60 °C	Rey Blanche	0.4 ± 0.01	4.03 ± 0.57	35.24 ± 4.5	42.5 ± 0.5
	70 °C		0.35 ± 0.02	3.13 ± 0.39	33.7 ± 2.7	37.71 ± 3.4
	80 °C		0.31 ± 0.01	2.68 ± 0.47	23.52 ± 2.2	30.5 ± 1.4

**Table 4:** Analysis of variance using GLM procedures.

Effect	Wilks’ λ value	F	Hypothesis df	Error df	Sig
Variety	.837	2.23	2	23	.130
Air flow rate	.081	130.26	2	23	p < 0.001
Temperature	.028	57.81	4	46	p < 0.001
Temperature * Air flow rate * Variety	.139	5.54	14	46	p < 0.001

### Determination of the characteristic drying curve

**Figure 7:**
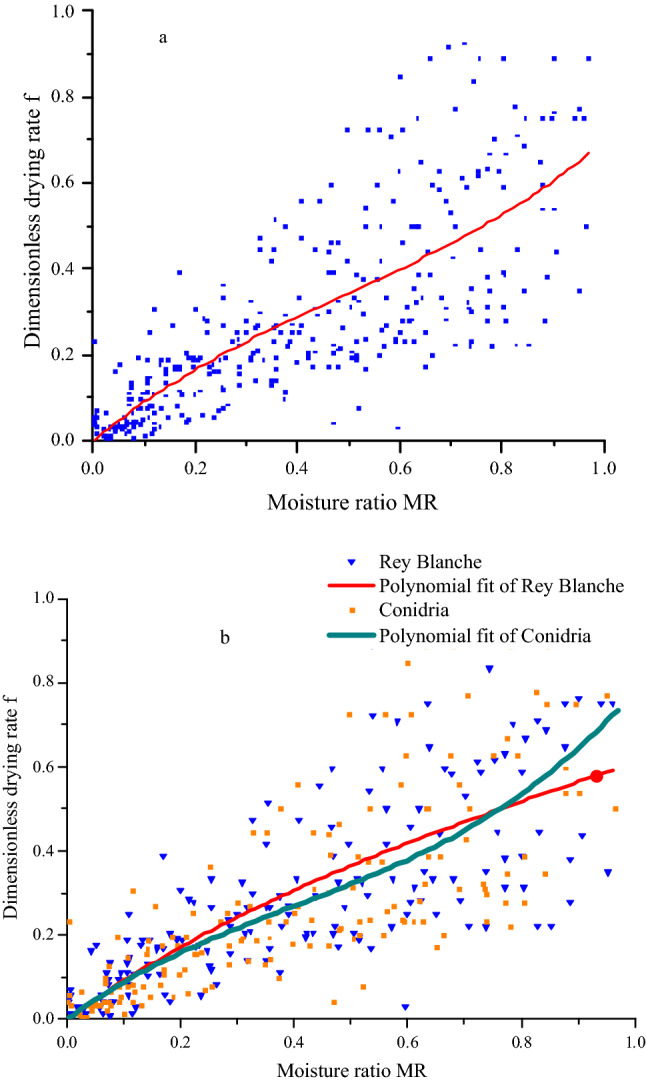
Characteristic drying curve of fig (**a**) and dimensionless drying rate versus moisture ratio of *Rey Blanche* and *Conidria* varieties (**b**).

Drying rate and time vary continuously according to the experiment conditions. Based on the Van Meel concept^59^ of the characteristic drying curve (CDC) may be exploited to establish a drying law for the samples from the experimental data. The principal interest of CDC is normalizing the kinetics of drying in a theoretical model to predict other drying curves under any aerothermal conditions, with regards to the sample initial water content and equilibrium moisture content. Indeed, to plot the CDC, it is required to gather all the data on a single curve using the non-linear optimization method of Levenberg Marquard. Figure 7a displays the dimensionless drying rate (f) as a function of the MR. The CDC was expressed in terms of the reduced moisture content as the following third-order polynomial function: f = 0.7945 MR-0.8774 MR2 + 0.6000MR3. Two criteria were used to evaluate fitting quality of the polynomial model, which were the standard error (SE = 0.1434) and the correlation coefficient (r = 0.9171).

The kinetics of moisture ratio versus dimensionless drying rate f of both fig varieties is presented in Fig. 7b. This curve determines the values of the initial water content and that of equilibrium, which are needed to describe the kinetics of drying at variables air-drying conditions.

The fig slices moistures ratios spatial pattern versus dimensionless drying rates attained at polynomial models for the two fig varieties and their respective correlation coefficients and standard deviations values were provided in Table 5. It is noteworthy that these curves displayed almost similar correlation coefficients (r), which were 0.9192 and 0.9174 for Rey Blanche and Conidria, respectively, whereas, the standard deviations were successfully 0.1449 and 0.1406.

**Table 5:** Polynomial equations of the characteristic drying equations for both tested fig varieties.

Varieties	CDE	r	SE
Rey Blanche	0.9544MR -0.5549MR^2^ + 0.2122MR^3^	0.9192	0.1449
Conidria	0.9810 MR -1.1576MR^2^ + 0.9582MR^3^	0.9174	0.1406

### Effective moisture diffusivity (D_eff_)

**Figure 8:**
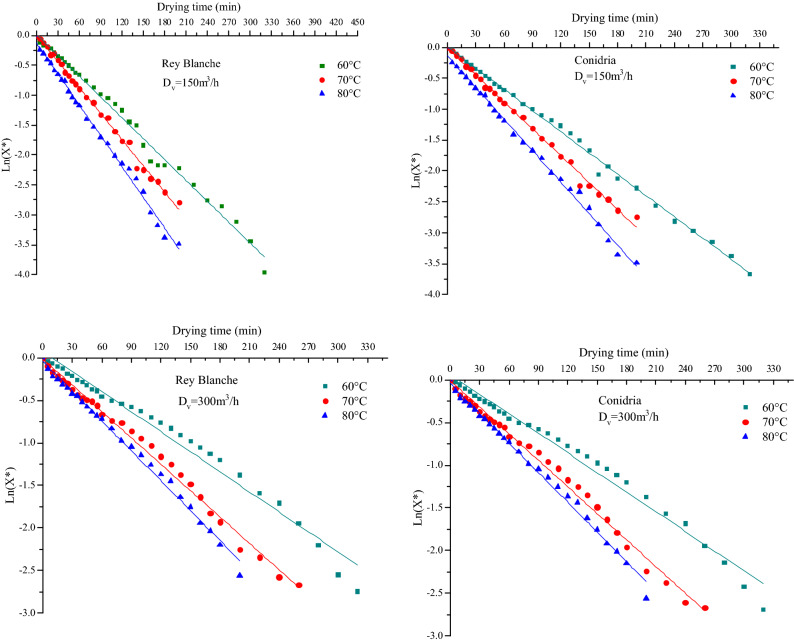
Plot of Ln(Y*) versus drying time in different drying air conditions for both fig varieties ‘Rey Blanche’ and ‘Conidria’.

D_eff_ is an important indicator that helps in designing and modeling of the mass transfer processes, such as drying or moisture adsorption during storage. It is mainly linked to the product’s temperature, the moisture content, and the structure. The D_eff_, is graphically determined using the logarithmic representation of the reduced moisture content X* as a function of the drying time. The plot of Ln(X*) versus drying period for different experimental sets was presented in Fig. 8. It illustrates the effect of temperature and velocity on the effective diffusion coefficient of fig slices. Thus, at a constant velocity level, D_eff_ increases continuously as the drying temperature increased. It is also noted that for both varieties, the D_eff_ increased subsequently as the drying air flow rate increases (Fig. 8). Table 6 presents D_eff_ values for both varieties at different experiment conditions. In general, D_eff_ varied between 1.9556 × 10^−9^ and 4.0511 × 10^−8^, that lie within the range 10^−8^ to 10^−12^ m^2^/s for drying of food materials. In general, when fig slices were dried at higher air temperature and air flow rate, increased heating energy systematically accelerates the activity of water molecules leading to higher moisture diffusivity^2^. These results were in agreement with those reported by^60^ on grape and^19^ on sweet cherry, who revealed that the moisture effective diffusivity increased proportionally to the drying temperature.

**Table 6:** Values of effective diffusivity of fig slices.

Variety	Air flow rate (m^3^/h)	Drying temperature (°C)	D_eff_ (m^2^/s)	r	SE
Rey Blanche	150	60	5.11 × 10^−09^	0.995	0.103
		70	1.79 × 10^−08^	0.997	0.061
		80	3.09 × 10^−08^	0.997	0.075
	300	60	1.95 × 10^−09^	0.989	0.111
		70	2.99 × 10^−09^	0.995	0.071
		80	9.97 × 10^−09^	0.996	0.058
Conidria	150	60	3.08 × 10^−09^	0.998	0.060
		70	2.41 × 10^−08^	0.99	0.059
		80	4.05 × 10^−08^	0.997	0.070
	300	60	2.20 × 10^−09^	0.995	0.072
		70	1.11 × 10^−08^	0.996	0.058
		80	1.66 × 10^−08^	0.991	0.098

### Energy of activation

**Figure 9:**
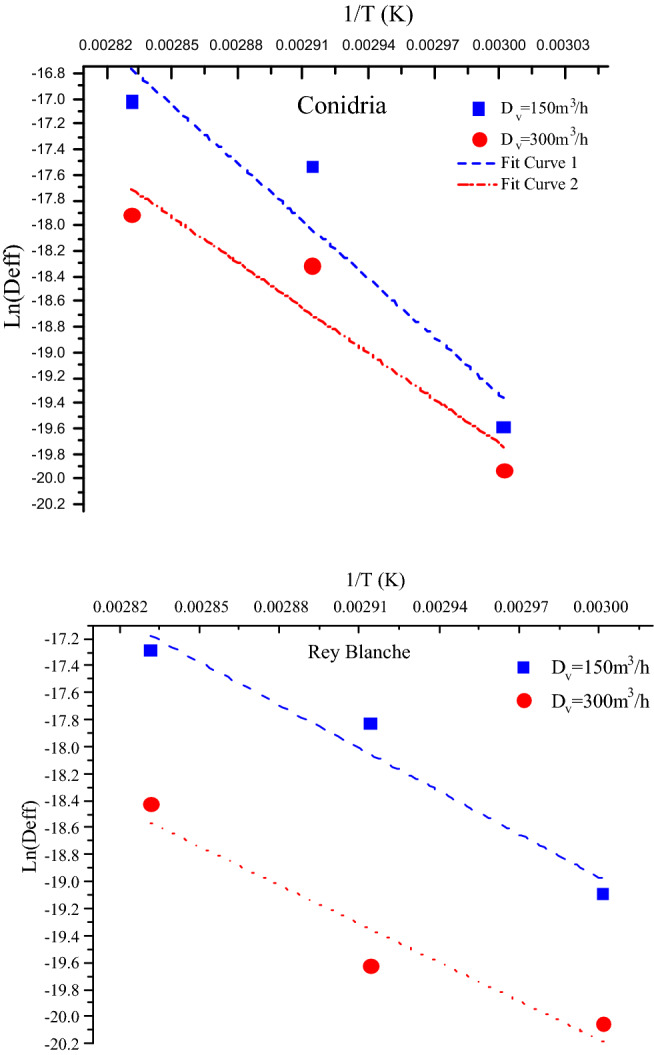
Ln(Deff) versus 1/T for both fig varieties at different air flows drying.

The energy of activation (Ea) was concluded by plotting Ln(D_eff_) versus 1/T, which the results were presented in the Fig. 9. Ea value was calculated in different drying experiments and listed in Table 7. The activation energy is considered as a great indicator of the required energy to remove moisture from the sample. Higher Ea value indicates high temperature sensitivity of diffusion coefficient. The obtained values of Ea were in the range of 4699.41 to 7502.37 kJ/kg. Ea required for water diffusion in “Rey Blanche” regardless the air velocity was lower than activation energy of “Conidria”. The coefficient of determination was slightly superior to 0.95 with a small standard error that was in average inferior to 0.6 for the two variables considered together (Table 7). In general, at the beginning of drying a little energy is required to remove moisture, and passing the time, the Ea increases because of the moisture resistance in fig tissues^30^. The results are comparable to those reported in similar studies on sweet cherry^19^, on banana slices^61^ and on peach and strawberry slices^62^.

**Table 7:** Energy of activation and related correlation coefficient for different levels of Air flows drying.

Sample	D_v_	Ea (kJ/kg)	r	SE
Rey Blanche	150	5240.20	0.9786	0.2691
	300	4699.41	0.9594	0.3369
Conidria	150	7502.37	0.9504	0.5986
	300	5886.17	0.9502	0.4711

### Modelling of fig drying curves

The samples moisture content at each drying temperature were converted afterwards to dimensionless moisture ratio and afterward fitted to nine empirical and non-empirical mathematical models (Table 2) to determine the statistics values, that give the best fit of models. The statistics of these models were estimated using non-linear regression analysis and summarized in Table 8. The correlation coefficient (r) was the primary criterion for choosing the model with the highest accuracy in drying curve prediction. Other than r, the mean square of the deviations (khi-square) between the calculated and predicted values for the models was used to determine the accuracy of the fit. Indeed, the model presenting the best prediction of fig drying curve was determined as the one with the highest r and the lowest of χ^2^. In fact, the r values ranged from 0.5977 to 0.9997 for ‘Conidria’ and from 0.6009 to 0.9997 for ‘Rey Blanche’, while χ^2^ were in the range of 1.13 × 10^−3^ to 0.2353 and 6.4 × 10^−3^ to 0.59 for ‘Conidria’ and ‘Rey Blanche’, respectively (Table 8). Among all the models tested, the modified Handerson and Pabis model provided the best prediction throughput resolution by displaying the highest r and the lowest value of χ^2^. In the literature, this model was successfully applied to pistachio^63^, kiwifruit^64^ and coconut^65^ to determine the drying curve.

**Table 8:** Statistical parameters obtained from all drying models for both fig varieties according to the drying temperature and air flow rate.

	Temperature (°C)	Conidria				Rey Blanche			
		D_v_ = 150		D_v_ = 300		D_v_ = 150		D_v_ = 300	
		r	χ^2^	r	χ^2^	r	χ^2^	r	χ^2^
Lewis	60	0.9993	0.0011	0.9975	0.0196	0.9979	0.0199	0.9972	0.0212
	70	0.9991	0.133	0.9946	0.0283	0.9983	0.0183	0.9943	0.029
	80	0.9897	0.038	0.9932	0.0304	0.9905	0.037	0.9928	0.0313
	Average	0.9960	0.0573	0.9951	0.0261	0.9955	0.0250	0.9947	0.0271
Page	60	0.9993	0.0114	0.9975	0.0198	0.9989	0.0202	0.9021	0.59
	70	0.9991	0.0133	0.9966	0.0230	0.9985	0.0172	0.9964	0.0234
	80	0.9976	0.01875	0.9975	0.0187	0.9976	0.0190	0.9969	0.0207
	Average	0.9986	0.0144	0.9972	0.0205	0.9983	0.0188	0.96513	0.2113
Wang and Singh	60	0.9757	0.0691	0.995	0.0283	0.9756	0.0684	0.995	0.028
	70	0.9736	0.072	0.9843	0.0490	0.9776	0.0673	0.9838	0.0497
	80	0.9231	0.1059	0.9821	0.0501	0.9262	0.1046	0.9828	0.0326
	Average	0.9574	0.0823	0.9871	0.0425	0.9598	0.0801	0.9872	0.0367
Logarithmic	60	0.9994	0.0114	0.9986	0.0150	0.9984	0.018	0.9986	0.0153
	70	0.9992	0.013	0.9976	0.0194	0.9986	0.0167	0.9974	0.0203
	80	0.9965	0.0233	0.9978	0.018	0.9967	0.0236	0.9976	0.0187
	Average	0.9983	0.0159	0.998	0.0174	0.9979	0.0194	0.9978	0.0181
Diffusion approximation	60	0.9994	0.0115	0.9981	0.0177	0.9979	0.0201	0.9974	0.0206
	70	0.9991	0.0136	0.9946	0.0293	0.9985	0.0175	0.9943	0.0301
	80	0.9897	0.0402	0.9993	0.0099	0.9905	0.0388	0.9928	0.0326
	Average	0.9960	0.0217	0.9973	0.0190	0.9956	0.0254	0.9948	0.0277
Midilli-Kucuk	60	0.7182	0.2273	0.7384	0.1985	0.7212	0.2234	0.7424	0.1982
	70	0.6944	0.2353	0.6824	0.2115	0.7031	0.2359	0.682	0.2116
	80	0.5977	0.2301	0.6093	0.2208	0.6009	0.2307	0.6127	0.2203
	Average	0.6701	0.2309	0.6767	0.2102	0.6750	0.23	0.6790	0.2100
Handerson and Pabis	60	0.9993	0.0114	0.9975	0.0199	0.9981	0.0191	0.9942	0.0215
	70	0.9992	0.0129	0.9973	0.0203	0.9985	0.0173	0.9971	0.021
	80	0.996	0.0244	0.9978	0.0174	0.9962	0.024	0.9976	0.0185
	Average	0.9981	0.0162	0.9975	0.0193	0.9976	0.0201	0.9963	0.02033
Modified Handerson and Pabis	60	0.9994	0.0122	0.9975	0.0214	0.9981	0.0204	0.9971	0.0232
	70	0.9992	0.0138	0.9974	0.0217	0.9985	0.0186	0.9971	0.0226
	80	0.9994	0.0095	0.9997	0.0066	0.9993	0.0108	0.9997	0.0064
	Average	0.99933	0.0118	0.9982	0.0165	0.9986	0.0166	0.9979	0.0174
Two term	60	0.9994	0.0116	0.9975	0.206	0.9985	0.0172	0.9977	0.0199
	70	0.9992	0.0133	0.998	0.1786	0.9985	0.0177	0.9979	0.0185
	80	0.9961	0.0254	0.9993	0.0102	0.9962	0.0249	0.9991	0.0141
	Average	0.9982	0.0167	0.9982	0.1316	0.9977	0.0199	0.9982	0.0175

**Figure 10:**
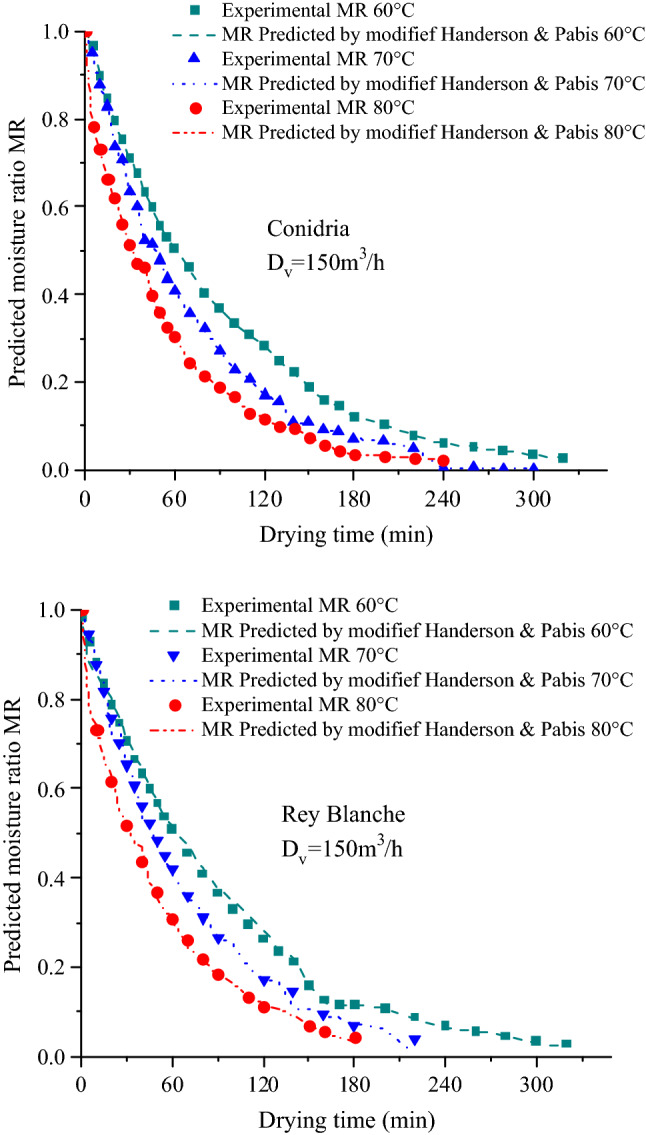
Experimental data of moisture ratio versus drying time fitted with modified Handerson and Pabis model at Dv = 150 m3/h.

**Figure 11:**
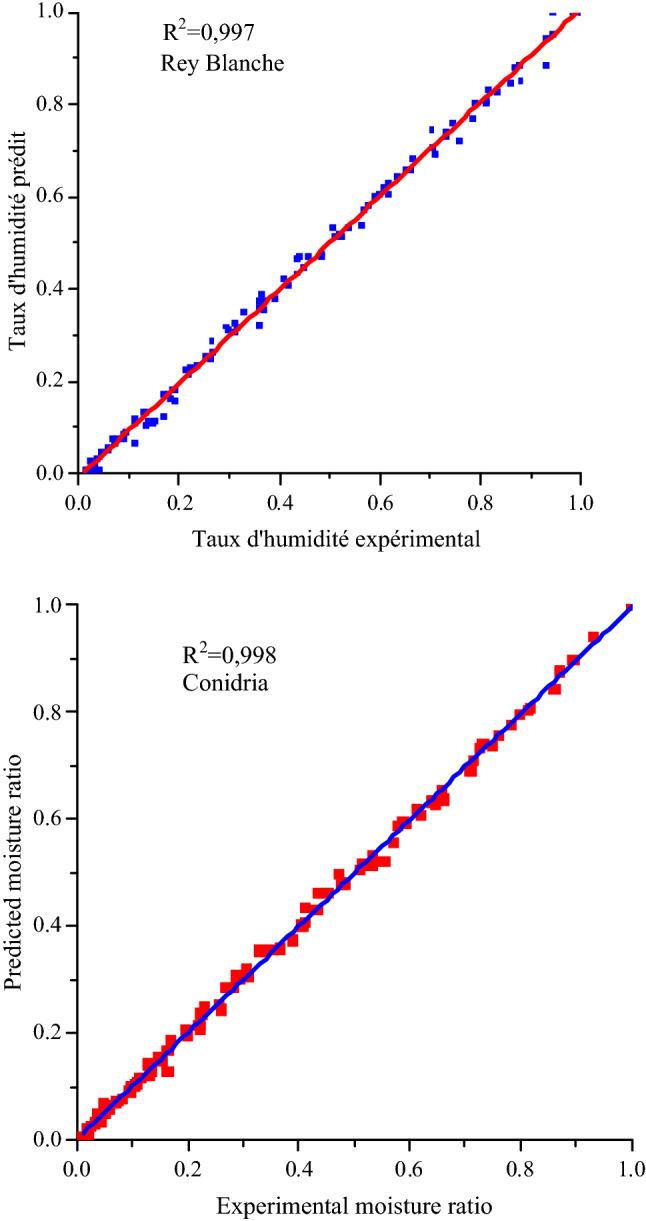
Comparison of experimental and predicted moisture ratio for both fig varieties by modified Handerson and Pabis model.

The experimental moisture ratios and those predicted by the selected model were plotted according to different air temperatures and flow rates in order to validate the model (Figs. 10 and 11). The fitting results demonstrates that Modified Handerson and Pabis prediction is suitable to describe the kinetics drying of fig slices. Figure 10 compared the temporal variation of experimental MR and those predicted by the selected model at D_v_ = 150 m^3^/h, where both curves are perfectly matching. In Fig. 11, the prediction using modified Handerson and Pabis model showed MR values banded along a straight line with a highly important value of the coefficient of determination, that generally exceeded 0.99, which attests the suitability of the selected model in describing the fig slices drying process.

### Total energy consumption in solar convective drying

**Figure 12:**
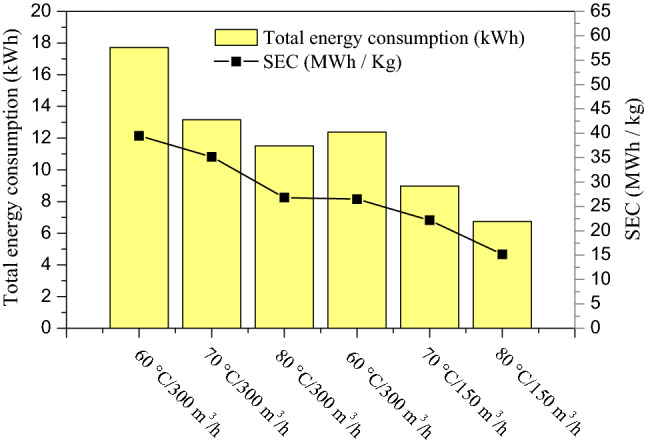
Energy consumption and specific energy consumption (SEC) during convection solar drying of fig slices at different aero-thermal conditions.

Figure 12. shows the total energy consumption in solar convection drying of fig at different air temperatures and flow rates. It was observed that the total energy consumption decreases as the air flow increases under the entire experimental temperature range. Likewise, at a constant air flow rate, the drying total energy consumption decreases substantially as the air temperature increases. That implies that the temperature increase leads to a substantial decrease of the drying time, which impacts significantly the dryer total energy consumption. These findings are in agreement with multiples studies previously published regarding several agri-foods products drying such as pomegranate arils, Russian olive, and chamomile^66–68^.

Figure 12, Made it possible to illustrate the specific energy consumption (SEC) values for the fig convective solar drying. Under all the experimental aerothermal conditions (temperatures of 60, 70 and 80 °C and air flow rate of 150 and 300 m^3^/h), the SEC values were obtained in the range of 13.12 and 44.55 MWh/kg. Similar results were described in a previous study on air drying of peppermint leaves^45^. Furthermore, it was observed that an increase in the air temperature implies a sharp decrease in the SEC. Moreover, an increase in the air flow rate also leads to a significant decrease in the specific energy consumption, for all air-drying temperatures. Comparable results were found on chamomile, berberis fruit, pomegranate arils, and peppermint leaves^45,66–69^.

### Energy efficiency

**Figure 13:**
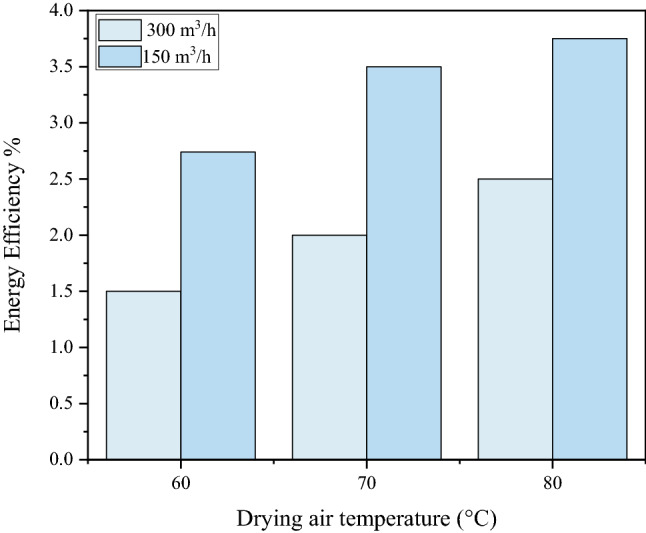
Energy efficiency for drying of fig slices at different drying air temperatures and flow rates.

The Fig. 13 displays the values of energy efficiency for multiple drying conditions of fig. The thermal efficiency represents the ratio between the amount of energy used for moisture elimination and the one supplied to the hybrid solar dryer. As it is described in the figure, the energy efficiency substantially decreases as the air flow rate and the drying temperature increase. According to this figure, the lowest energy efficiency was about 1.54%, and was found in the combination of a temperature of 60 °C and a velocity of 300 m^3^/h, while the maximum value was around 3.98%, and was achieved in the combination of 80 °C and 150 m^3^/h. The fig drying data generated using a solar convective dryer shows similar trends in comparison to the thin drying air convection of peppermint leaves (3.5- 5.3%) and convective drying of chamomile (1.9- 6.7%). The energy efficiency values obtained are in agreement with those found for most convection dryers^45,68^.

Fundamentally, it was stated that the final moisture in biological products generally requires higher drying energy than the initial moisture, and the preparation of the samples prior to drying such as osmotic treatment^70^, application of some antibrowning agents^71,72^ affects the thermal efficiency. These factors make it difficult to compare with the thermal efficiencies of other solar dryers reported in previous^73^.

## Conclusion

This work is an experimental study of thin-layer solar drying of two fig varieties growing in Morocco. It was conducted during highest solar radiation period in one of the highest insolation cities in Morocco. The experimental design combined three air drying temperatures (60, 70 and 80 °C) and two drying velocities (150 and 300 m^3^/h). The results displayed that the impact of drying air temperature on the moisture pattern versus drying time on the fig drying process is more important as compared to the influence of the air-drying flow rate. Nine drying models, most used in the literature, were fitted to the experimental data. Modified Handerson and Pabis model showed the best prediction of fig slices drying curve. Statistical analysis using GLM procedures showed that the time required form drying could be significantly shortened by combining higher temperature (80 °C) and air velocity (300 m^3^/h), of which the sample physical traits were significantly important compared to other combinations and the energy consumption was significantly low. The ANOVA model using GLM procedure showed that the behavior of studied varieties was statistically similar. As the entire fig drying occurs in the falling-rate period, the second Fick’s law was then used to calculate D_eff_. The increase in drying temperature at a constant air flow rate increased the effective moisture diffusivity described using Arrhenius equation. This study was first thin layer convective drying conducted on fig in Morocco. It allowed to determine the characteristic drying curve equation that is essential in dimensioning an adequate and efficient fig solar dryer. It was undertaken to understand the fig thin-layer drying, evaluate and save energy consumption of the drying process. This study clearly shows that the air temperature as well as the velocity had a large impact on the overall energetic performance of the hybrid dryer, with a substantially low electrical power requirements, as these factors increase. The results suggest that the solar energy can be an effective renewable source in biological products drying process, which can become the most efficient and feasible way out to deal with the increasing energy demand and supply gap.

## Abbreviations

CDC: Characteristic drying curve
CDE: Curve drying equation
Dv: Drying volume flow rate (m^3^/h)
d.b: Dry basis
$\left( { - \frac{dM}{{dt}}} \right)_{0}$: Initial drying rate (kg water/(kg dry matter.min))
$\left( { - \frac{dM}{{dt}}} \right)_{t}$: Drying rate at a specific drying time (kg water/(kg dry matter.min))
f: Relative drying rate (dimensionless)
M: Moisture content (% dry weight)
M_0_: Initial moisture content (% dry weight)
M_e_: Equilibrium moisture content (% dry weight)
Mf: Final moisture content (kg water/kg dry weight)
MR: Moisture ratio
SE: Standard error
R: Correlation coefficient
w.b: Weight basis
GLM: General linear model
ΔE: Total color difference
χ^2^: Chi square
i: Observation number
BTU: British thermal unit
ft: Foot
W: Watt
ABS: Absolute humidity
HR: Relative humidity
