# Author Correction: Kinetics, energy efficiency and mathematical modeling of thin layer solar drying of figs (*Ficus carica* L.)

**DOI:** 10.1038/s41598-021-01688-3

**Published:** 2021-11-09

**Authors:** Lahcen Hssaini, Rachida Ouaabou, Hafida Hanine, Rachid Razouk, Ali Idlimam

**Affiliations:** 1 National Institute of Agricultural Research (INRA), Rabat, Morocco; 2 Department of Chemistry, Faculty of Science Semlalia, Cadi Ayyad University, B.O. 2390, 40000 Marrakesh, Morocco; 3 Laboratory of Bioprocess and Bio‑Interfaces, Faculty of Science and Technics, BO 523, Beni-Mellal, Morocco; 4 Laboratory of Solar Energy and Medicinal Plants, Teacher’s Training College, Cadi Ayyad University, BP 2400, Marrakesh, Morocco

Correction to: *Scientific Reports* 10.1038/s41598-021-00690-z, published online 28 October 2021

The original version of this Article contained errors in the Abstract.

“First convectional thin layer drying of two fig (*Ficus carica* L.) varieties growing in Moroccan, using partially indirect convective dryer, was performed. The experimental design combined three air temperatures levels (60, 70 and 80 °C) and two air-flow rates (150 and 300 m^3^/h).”

now reads:

“First convectional thin layer drying of two fig (*Ficus carica* L.) varieties growing in Morocco, using partially indirect convective dryer, was performed. The experimental design combined three air temperature levels (60, 70 and 80 °C) and two air-flow rates (150 and 300 m^3^/h).”

“The average activation energy was ranged between 4699.41 and 7502.37 kJ/kg. It raised proportionally with the air flow velocity, and the same pattern were observed for effective moisture diffusivity regarding drying time and velocity.”

now reads:

“The average activation energy ranged between 4699.41 and 7502.37 kJ/kg. It raised proportionally with the air flow velocity, and the same patterns were observed for effective moisture diffusivity regarding drying time and velocity.”

“Likewise, the energy efficiency was greater (3.98%) higher in drying conditions.”

now reads:

“Likewise, the energy efficiency was greater (3.98%) in drying conditions.”

The original Article has been corrected.

